# Targeting Alzheimer’s disease with a Geroscience approach

**DOI:** 10.1002/alz.093259

**Published:** 2025-01-03

**Authors:** Martin Darvas

**Affiliations:** ^1^ University of Washington School of Medicine, Seattle, WA USA

## Abstract

**Background:**

A drug cocktail targeting different processes of aging was tested in an aging mouse model of Alzheimer’s disease (AD) neuropathologic change as an intervention to improve behaviors corresponding to cognitive dysfunction in AD.

**Method:**

A cocktail of acarbose/rapamycin/phenylbutyrate or a control treatment was administered (medicated vs. non‐medicated chow) chronically to 22 months‐old mice that received viral vector injections to induce amyloid and tau pathology in the hippocampus at 24 months of age. At 27 months of age motor, anxiety and cognitive behaviors were measured using open field, y‐maze and contextual‐fear conditioning tests.

**Result:**

The percentage of spontaneous alternations in the y‐maze of mice with hippocampal injection of viral vectors to induce amyloid and tau pathology (amyloid‐tau group) was significantly reduced when compared to mice that received hippocampal injection of control viral vectors (sham group). Further, the percent of time spent freezing in the fear‐conditioning apparatus of mice from the amyloid‐tau group was significantly reduced when compared to sham‐control mice. Treatment with the drug cocktail improved both spontaneous alternations in the y‐maze and time spent freezing in the fear‐conditioning apparatus in mice from the amyloid‐tau group.

**Conclusion:**

The drug cocktail of acarbose/rapamycin/phenylbutyrate reduced the negative effects of hippocampal amyloid and tau pathology on two measures of cognitive function.

